# Application of a *VP4/VP2*-inferred transmission clusters in estimating the impact of interventions on rhinovirus transmission

**DOI:** 10.1186/s12985-022-01762-w

**Published:** 2022-03-04

**Authors:** Kim Tien Ng, Liang Jie Ng, Xiang Yong Oong, Jack Bee Chook, Kok Gan Chan, Yutaka Takebe, Adeeba Kamarulzaman, Kok Keng Tee

**Affiliations:** 1 Department of Medicine, Faculty of Medicine, University of Malaya, Kuala Lumpur, Malaysia; 2 Infectious Diseases Translational Research Program, Yong Loo Lin School of Medicine, National University of Singapore, Singapore, Singapore; 3 Department of Microbiology and Immunology, Yong Loo Lin School of Medicine, National University Health System, National University of Singapore, Singapore, Singapore; 4 Faculty of Information Science and Technology, Multimedia University, Melaka, Malaysia; 5 Department of Medical Microbiology, Faculty of Medicine, University of Malaya, Kuala Lumpur, Malaysia; 6 School of Medical and Life Sciences, Sunway University, Bandar Sunway, Selangor Darul Ehsan Malaysia; 7 Division of Genetics and Molecular Biology, Institute of Biological Sciences, Faculty of Science, University of Malaya, Kuala Lumpur, Malaysia; 8 AIDS Research Center, National Institute of Infectious Diseases, Toyama, Shinjuku-ku, Tokyo, Japan

**Keywords:** Acute respiratory tract infection, Rhinovirus, *VP4/VP2* gene, Transmission clusters, Interventions

## Abstract

**Background:**

Despite the clinical burden attributable to rhinovirus (RV) infections, the RV transmission dynamics and the impact of interventions on viral transmission remain elusive.

**Methods:**

A total of 3,935 nasopharyngeal specimens were examined, from which the *VP4/VP2* gene was sequenced and genotyped. RV transmission clusters were reconstructed using the genetic threshold of 0.005 substitutions/site, estimated from the global *VP4/VP2* sequences. A transmission cluster is characterized by the presence of at least two individuals (represent by nodes), whose viral sequences are genetically linked (represent by undirected edges) at the estimated genetic distance threshold supported by bootstrap value of ≥ 90%. To assess the impact of facemask, pleconaril and social distancing on RV transmission clusters, trials were simulated for interventions with varying efficacy and were evaluated based on the reduction in the number of infected patients (nodes) and the reduction in the number of nodes-connecting edges. The putative impact of intervention strategies on RV transmission clusters was evaluated through 10,000 simulations.

**Results:**

A substantial clustering of 168 RV transmission clusters of varying sizes were observed. This suggests that RV disease burden observed in the population was largely due to multiple sub-epidemics, predominantly driven by RV-A, followed by RV-C and -B. No misclassification of RV species and types were observed, suggesting the specificity and sensitivity of the analysis. Through 10,000 simulations, it was shown that social distancing may be effective in decelerating RV transmission, by removing more than 95% of nodes and edges within the RV transmission clusters. However, facemask removed less than 8% and 66% of nodes and edges, respectively, conferring moderate advantage in limiting RV transmission.

**Conclusion:**

Here, we presented a network-based approach of which the degree of RV spread that fuel disease transmission in the region was mapped for the first time. The utilization of RV transmission clusters in assessing the putative impact of interventions on disease transmission at the population level was demonstrated.

## Background

Rhinoviruses (RVs), the primary viral etiology of acute respiratory tract infections, continue to contribute significantly to global health burden through increased medical expenses and loss of productivity [1]. While studies have reported that infectious diseases such as influenza, varicella, herpes zoster, rubella and measles are suppressed [2–4], RV infections remains common during the relentless fight against the unceasing coronavirus disease (COVID-19) pandemic by severe acute respiratory syndrome coronavirus 2 (SARS-CoV-2) [5]. On average, RV causes 2–4 episodes of respiratory infections in adults and 8–12 episodes in children each year [6]. More importantly, reports have highlighted the involvement of RV in lower respiratory tract infections [7, 8]. Furthermore, several studies have demonstrated that individuals with underlying respiratory conditions may experience severe RV-induced complications, as observed in the exacerbation of asthma, chronic obstructive pulmonary disease (COPD) and cystic fibrosis [9–11].

Despite its disease burden, factors that drive the virus transmission remain unclear. Investigation based on viral sequence data have unraveled the potential role of transmission clusters in fueling the disease expansion [12]. For instance, in a spatial analysis on the Middle East respiratory syndrome coronavirus (MERS-CoV) outbreaks, it has been demonstrated that the persistence of MERS-CoV infection was established through the presence of transmission clusters [13]. Likewise, such finding was observed in other viral infection such as the 2019 Ebola virus outbreaks [14]. Although RV is commonly associated with respiratory tract infections, the transmission pattern of RV remains insufficiently explored. Importantly, the risk of continual emergence of transmission clusters in the absence of effective interventions may result in expanded and sustained viral transmission, as observed in the current COVID-19 pandemic [15].

Treatment of RV infection remains supportive and non-specific, with no licensed vaccine or approved antiviral therapy available [16]. The challenge may be compounded by the presence of three confirmed RV and a provisional fourth RV species [17] that diverged further into more than 160 genetically distinct types with extensive sequence variability at the antigenic sites [18, 19]. Although non-pharmaceutical interventions (e.g., social distancing and facemask) are available to mitigate disease expansion [20, 21], the effectiveness of such interventions in controlling viral transmission in the population is difficult to measure and remains elusive. To date, the putative impact of interventions on viral transmission has only been inferred for the human immunodeficiency virus using a network-based analysis [22].

Here, based on the viral genetic diversity, we investigated the dynamics of RV transmission clusters in a population from individual *VP4/VP2* gene, followed by simulation of the impact of intervention strategies on RV infections at the population level. Importantly, the integration of the proof-of-concept analysis into conventional molecular epidemiological surveillance may enable a near real-time investigation on virus spread and control.

## Methods

### Ethics statement, study subjects and specimens

This study was approved by the University Malaya Medical Centre Medical Ethics Committee (MEC890.1). Standard, multilingual consent forms validated by the Medical Ethics Committee were used. Written informed consent was obtained from all study subjects. All experiments were performed in accordance with approved guidelines and regulations. Consenting outpatients who presented with symptoms of acute upper respiratory tract infections were enrolled at the primary care clinics, University Malaya Medical Centre, Kuala Lumpur, Malaysia between February 2012 and May 2014. Respiratory specimens in the form of nasopharyngeal swabs were collected in universal transport medium using standardized method. The specimens were transported to the laboratory and stored at − 80 °C before further processing.

### Molecular detection of RV

Total viral nucleic acid was extracted from nasopharyngeal specimens using the NucliSENS easyMAG automated nucleic acid extraction system (bioMérieux, Marcy I'Etoile, France), as described in the manufacturer's protocol. The specimens were screened for viral pathogens using the xTAG Respiratory Viral Panel (RVP) *FAST* Assay (Luminex Molecular, Toronto, Canada) and analysed using the Luminex’s proprietary Universal Tag sorting system on the Luminex 200 IS platform (Luminex Corp, Texas, USA). Specimens positive for human enteroviruses were further confirmed through PCR amplification and direct sequencing of the *VP4/VP2* gene using primers described previously [23]. The contiguous nucleotide sequences generated by an ABI Prism 3730xl DNA analyzer (Applied Biosystems, USA) were assembled and codon aligned.

To determine the types of these circulating RV, neighbour-joining trees were first reconstructed based on an updated and comprehensive list of global *VP4/VP2* sequence data (*n* = 3,397), retrieved from GenBank (accessed on March 2016), using Kimura 2-parameter model implemented in MEGA [24]. The statistical robustness of the branching orders was assessed by bootstrap analysis of 1000 replicates.

### Estimation of patristic distance and reconstruction of RV transmission clusters

Communicable diseases are often disseminated through close contact between the infected and susceptible individuals. To reconstruct RV transmission clusters, the genetic distance threshold is first determined between the lowest and the highest value of the inter- and intra-patient patristic distances (measured in nucleotide substitutions per site), respectively [25]. RV sequences from different patients with a patristic distance lower than the estimated threshold, as a measure of genetic relatedness and transmission linkages of the infecting RV strains, will be identified and grouped as transmission cluster. In acute respiratory tract infection, accurate estimation of intra-patient viral genetic distance was particularly challenging due to difficulty in sampling. Therefore, the most plausible threshold value was determined from the lowest 95% confidence interval of the lower 0.05 percentile of the inter-patient genetic distance [25], as calculated from the global *VP4/VP2* gene sequences of various RV types.

With the genetic distance threshold estimated, the transmission clusters were reconstructed from all newly sequenced Malaysian RV *VP4/VP2* gene sequences based on the Tamura-Nei 93 (TN93) pairwise distance performed using a custom script in Python (release 3.2.6), with bootstrap analysis of 1000 replicates [25] and visualized using Gephi (version 0.9.) [26]. Although previous studies have addressed the population dynamics and transmission clusters of the respiratory viruses [12, 27], such features in RV infections have never been reported. Therefore, similar classification of transmission clusters was adopted, in which, a transmission cluster is characterized by the presence of at least two individuals (represent by nodes), whose viral sequences are genetically linked (represent by undirected edges) at the estimated genetic distance threshold supported by bootstrap value of ≥ 90%. In general, clusters were described as dyads if they contain two nodes, and networks if more than 2 nodes were observed. On the other hand, those that do not form clusters are termed as singleton.

### Investigating the impact of interventions on RV transmission clusters through simulation

Preliminary investigations (either in vitro, pseudo-steady conditions or simulations) on antiviral therapy (e.g., pleconaril against enteroviruses) and other non-pharmaceutical intervention strategies (e.g., facemask and social distancing) have demonstrated moderate-to-high efficacy in inhibiting viral transmission [28, 29]. However, the efficacy of these interventions in preventing viral transmission at the population level remains unclear. To investigate the population-level impact of intervention strategies through simulation, the putative transmission clusters inferred from the *VP4/VP2* gene at individual level was utilized as baseline. To quantitate the impact of interventions (facemask, oral pleconaril and social distancing) on RV transmission clusters, trials were simulated for (a) interventions with varying efficacy (0% through 100%), that corresponds to the decline in the number of infected patients (nodes) and (b) interventions with varying efficacy (0% through 100%), that corresponds to the reduction in the number of edges connecting to the nodes. For each setting (intervention efficacy), 10,000 trials were simulated. Briefly, the aligned RV *VP4/VP2* sequences (accession numbers KY093077 – KY094053) in.fasta format were loaded and the procedure implemented in Python custom script was performed in less than 30 min on Linux Operating System. The scripts, Clustering.py for the construction of transmission networks and DistanceFilter.py for the assessment of interventions have been made available online and accessible at https://github.com/nglj93/Transmission-Network. The procedure was implemented in Python using custom script. The numbers of nodes and edges removed at varying intervention efficacy were plotted, from which the efficacy of an intervention required to reduce 50% proportion of the nodes (NP_50_) and edges (EP_50_) were estimated. From the plot, the impact of existing intervention strategies on viral transmission at population level were extrapolated.

## Results

### Prevalence and distribution of RV species among patients with acute respiratory infection

Between February 2012 and May 2014, a total of 3,935 consenting outpatients presented with symptoms of acute respiratory tract symptoms were recruited, of whom 51.1% (2,009/3,935) were positive for at least one viral pathogen in the multiplex respiratory virus panel screening assay. Among the 2,009 subjects, 976 (48.6%) were positive for RV. Neighbour-joining phylogenetic analysis of the *VP4/VP2* gene showed the predominance of RV-A (48.5%; 473/976), followed by RV-C (38.1%, 372/976) and RV-B (13.4%, 131/976) [12]. Additional information (patients demographic and symptoms severity) are published online [12]

### Genetic distance threshold and transmission clusters

**Fig. 1:**
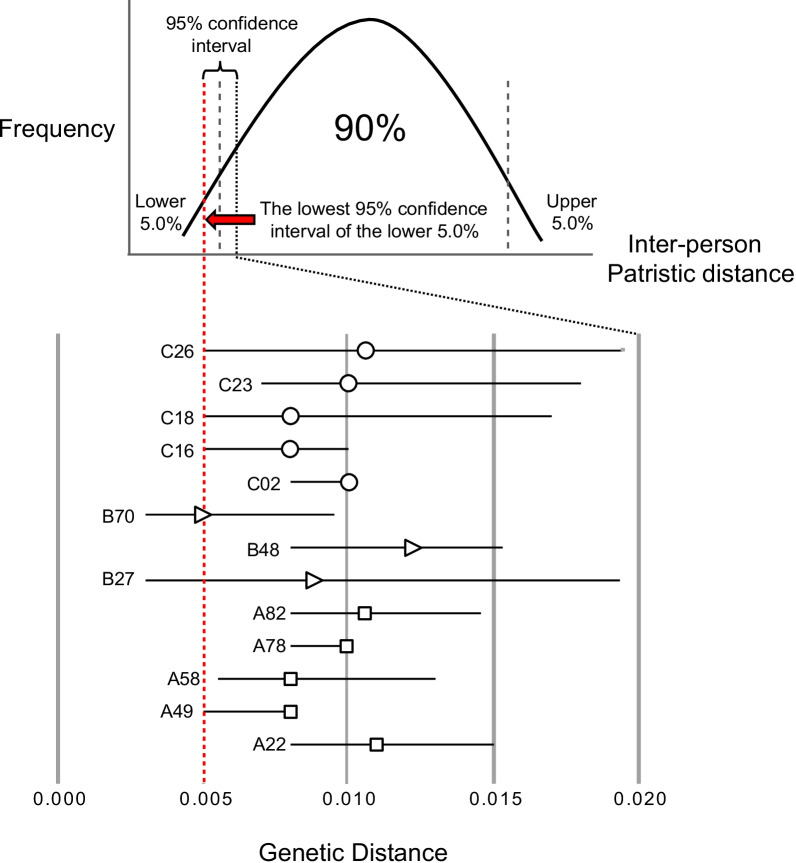
The estimation of genetic distance threshold for reconstruction of RV transmission clusters. The genetic distance threshold (measured in nucleotide substitutions per site) was determined from the lowest 95% confidence interval of the lower 0.05 percentile of the inter-patient patristic distance, as calculated from the global RV *VP4/VP2* gene. In the present study, the genetic threshold was estimated at 0.005 substitutions/site across RV-A, -B and -C (indicated in red dotted line)

To investigate the dynamics of RV spread in the study population, the transmission clusters were reconstructed based on the pairwise distances estimated from the global RV *VP4/VP2* gene sequences, using the TN93 model. The distance threshold (the lowest 95% confidence interval of lower 0.05 percentile of the inter-patient patristic distance) for reconstruction of RV transmission clusters was estimated at 0.005 substitutions/site (across RV-A, -B, and -C) (Fig. 1).

**Fig. 2:**
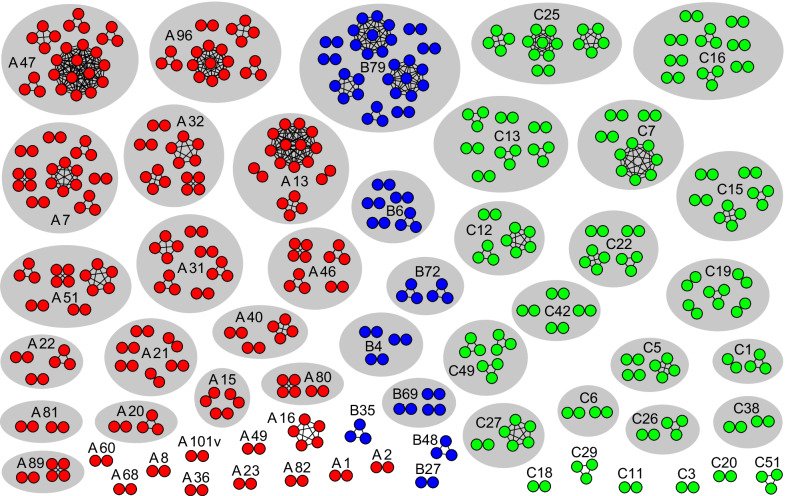
Transmission clusters of RV among patients presented with symptoms of acute respiratory tract infection in Kuala Lumpur, Malaysia between 2012 and 2014. With an estimated genetic threshold of 0.005 substitutions/site, RV transmission clusters were inferred from 976 newly sequenced *VP4/VP2* sequences based on Tamura-Nei 93 (TN93) pairwise distance performed using a custom script in Python (release 3.2.6), with bootstrap analysis of 1000 replicates. A total of 168 RV transmission clusters of varying size (2–13 nodes per cluster), involving 57 different RV types were identified. In general, more transmission clusters were observed in RV-A, followed by RV-C and -B. In the present study, transmission cluster is characterized by the presence of at least two individuals (represent by nodes), whose viral sequences are genetically linked (represent by undirected edges) at 0.005 substitutions/site supported by bootstrap value of ≥ 90%. Transmission clusters were described as dyads if they contain two nodes, and networks if more than 2 nodes were observed. Nodes are colour-coded in accordance with RV species

With this estimated genetic distance threshold, RV transmission clusters were inferred from 976 newly generated *VP4/VP2* sequences, yielding a total of 168 RV transmission clusters of varying size (2–13 nodes per clusters), involving 57 genetically distinct RV types (27 RV-A, 8 RV-B, and 22 RV-C types) (Fig. 2). In general, more RV-A transmission clusters (45.2%, 76/168) were observed, comprising of 43 dyads and 33 networks. In addition, a total of 67 (39.9%) RV-C clusters (42 dyads and 25 networks) and 25 (14.9%) RV-B clusters (16 dyads and 9 networks) were identified. To test the reliability of the genetic distance threshold (0.005 substitutions/site), changes in the total number of nodes and edges observed within the clusters were estimated under a range of genetic distances (0.001 through 0.025 substitutions/site). It was shown that the total number of nodes (n = 479) and edges (n = 665) within the transmission clusters were consistent at 0.001 through 0.005 substitutions/site, suggesting that the 0.005 substitutions/site threshold did not underestimate the total number of nodes and edges observed in this dataset (Fig. 2). Importantly, no misclassifications of RV species and types were observed, highlighting the specificity and sensitivity of the analysis. In contrast, the number of nodes and edges was predictably higher (overestimated) when the genetic distance threshold was raised beyond 0.005 substitutions/site.

### Putative impact of interventions on RV transmission dynamics

**Fig. 3:**
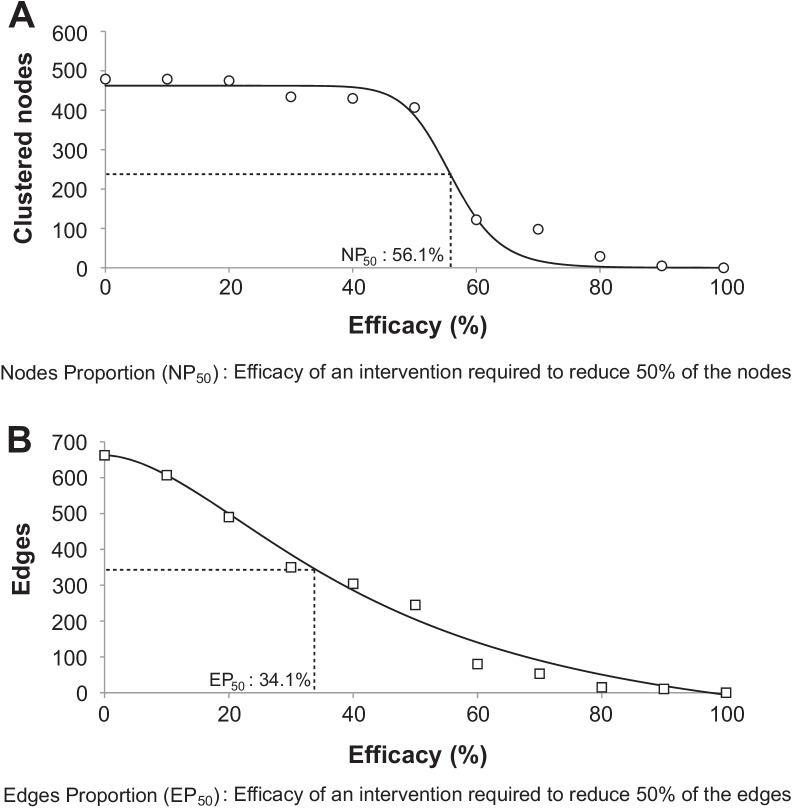
The probable impact of an intervention with varying efficacy on the dynamics of RV transmission. **A** It is shown that the total number of nodes is inversely associated with the efficacy of an intervention. It is hypothesized that an intervention requires a minimum efficacy of 56.1% to remove 50% of the nodes (NP_50_) within RV transmission clusters. **B** Similar inversed association was observed between the total number of edges and the efficacy of an intervention, in which a minimum efficacy of 34.1% is required to remove 50% of the edges (EP_50_) within RV transmission clusters. For each setting (intervention efficacy), 10,000 trials were simulated. The procedure was implemented in Python (release 3.2.6) using custom script

In this analysis, the efficacy of an intervention strategy was measured by means of a random reduction in the number of nodes and edges in the baseline transmission clusters. To investigate the impact of intervention on the nodes and edges within the clusters, varying degree of efficacy (0% through 100%) were simulated at 10,000 intervention trials. As anticipated, the total number of nodes and edges was inversely associated with the increasing efficacy of an intervention strategy (Fig. 3).

It was determined that an intervention strategy requires an efficacy of at least 56.1% and 34.1% to remove 50% proportion of the nodes (NP_50_) and edges (EP_50_) within the transmission clusters, respectively. Next, the impact of existing intervention strategies on viral transmissions at population level was extrapolated. It was plotted that facemask, with an efficacy of 45% [29] was able to eliminate only 7.3% (34.9/479) and 65.4% (434.6/665) of nodes and edges within RV transmission clusters, respectively (Table 1).

**Table 1:** The extrapolated impact of facemask, pleconaril and social distancing on RV transmission dynamics at the population level

Interventions	Efficacy level (%)	No. of nodes			No. of edges		
		Before intervention	After intervention^†^	Removed (%)	Before intervention	After intervention^†^	Removed (%)
Facemask	45^a^	479	444.1	34.9 (7.3)	665	230.4	434.6 (65.4)
Pleconaril	87^b^	479	24.9	454.1 (94.8)	665	9.2	655.8 (98.6)
Social distancing	90^c^	479	24.6	454.4 (94.9)	665	0.3	664.7 (99.9)

^a^Lai et al.[29]

^b^Pevear et al.[30]

^c^Glass et al.[31]

^†^Estimated through 10,000 simulation trials

On the other hand, unlicensed viral capsid-binding pleconaril, with a previously reported efficacy of approximately 87% [30] removed 94.8% (454.1/479) of nodes and 98.6% (655.8/665) of edges within RV transmission clusters [29]. Lastly, social distancing with a previously reported hypothetical efficacy of approximately 90% [31] eradicated 94.9% (454.4/479) and nearly 100% (664.7/665) of nodes and edges, respectively.

## Discussion

Acute respiratory tract infection is one of the most common infectious diseases in humans, contributing to approximately 15% of deaths due to all causes among children less than 5-year-old and responsible for over 4 million of annual deaths worldwide [1]. Although several viruses have been implicated in acute respiratory infections, the prevalence of RV is frequently observed [32]. Importantly, the involvement of RV in fatal respiratory outbreaks and the lower respiratory compartment resulting in severe respiratory conditions such as pneumonia, bronchiolitis, chronic bronchitis and exacerbation of asthma have become increasingly evident [10, 11, 33]. Despite the clinical burden, the transmission pattern of RV and the effectiveness of interventions in decelerating viral transmission have not been reported to date.

The dissemination and expansion of infectious diseases are primarily traced through passive and active surveillance that largely relies on the cooperation of the healthcare providers to report the cases, and deliberate search for cases through population survey [34, 35]. However, these resource-intensive methods are not without setbacks that could compromise the quality of the surveillance data, due to self-reported inaccuracies and data incompleteness. Interestingly, several studies have demonstrated the importance of molecular epidemiological surveillance in elucidating the epidemic linkages within a population [13, 25], thereby establishing a new frontier in delineating viral transmission dynamics.

In the present study, a network-based approach of which the degree of RV spread that fuel disease transmission in the region was mapped for the first time, using a programming language-based approach. With the TN93 pairwise distance estimate of the *VP4/VP2* gene (Fig. 1), the transmission clusters of RV-A, -B and -C were inferred. Based on previously described criteria of transmission clusters [25], a substantial clustering pattern (49.1%, 479/976 of Malaysian RV *VP4/VP2* sequences) involving numerous transmission clusters was identified (Fig. 2). This finding highlight that the RV disease burden observed between February 2012 and May 2014 in Kuala Lumpur was largely contributed by the presence of multiple sub-epidemics, predominantly driven by RV-A and -C. In the absence of effective interventions, the continuous emergence of RV transmission clusters plays an important role in disease persistence and expansion in the population.

The utilization of (passive and active) surveillance data in conceptualized framework [31] or computational models [36] to decipher the effectiveness of interventions in repressing the spread of respiratory virus within a community has been previously demonstrated. However, due to the nature of surveillance data [31, 36], the effectiveness of interventions in mitigating viral transmission at the population level remains to be ascertained. In the present study, which was established based on the large-scale molecular surveillance data, new parameters namely the NP_50_ and EP_50_ were proposed as a potential indicator that implies the effectiveness of an intervention. Through computational simulations, the NP_50_ and EP_50_ were estimated (Fig. 3), below which an intervention may be deemed less effective in controlling RV transmission at the population level.

In a prospective trial, the implementation of mask-wearing policy has been shown to reduce parainfluenza virus-associated respiratory illnesses [37]. However, the study was only limited within a hospital, highlighting the effects of mask-wearing on viral spread in the wider population remains debatable. Importantly, present study highlighted that facemask, with a reported efficacy of 45% [29], confers moderate advantage in limiting RV transmission clusters at the population level. This finding suggests that facemask may be of value in providing protections against respiratory viruses, especially among individuals at risk in controlled settings (i.e., hospitals). Social distancing is another commonly implemented public health non-pharmaceutical intervention strategy to minimize viral transmission in the time of disease epidemics, as observed in the recent COVID-19 pandemic [21]. Here, it has been shown that, social distancing is highly effective in controlling viral transmission and suppressing the expansion of RV transmission clusters. Such estimation is in line with previously reported findings that indicated social distancing can hypothetically reduce the influenza attack rate by at least 90% [28], suggesting that such strategy could be effective in providing defense against virus transmission in the absence of vaccine or antiviral therapy [38]. Although such strategy provides the highest level of protection, the implementation of social distancing can be challenging because the high level of compliance during the period of epidemic is the mainstay to the success of such strategy, as observed in recent COVD-19 pandemic [39].

Pleconaril is a capsid-binding antiviral that interferes the viral interaction with host cellular receptor, thereby inhibiting viral replication [30]. In a placebo-controlled trial, it has been shown that early pleconaril treatment was associated with a rapid loss of RV and heightened symptoms relief effects, highlighting its high therapeutic efficacy against RV-induced community-acquired respiratory infections [30]. Likewise, in the present network-based extrapolation, similar effect of pleconaril was reported, as reflected in a significant reduction in the number of nodes and edges (≥ 95%) within RV transmission clusters at population level. Despite the promising therapeutic impact, pleconaril remains unlicensed and its usage has been hindered by its pharmacodynamics (e.g., drug side effects and drug interactions), as observed in preliminary randomized trial [40].

In the present study, the potential use of transmission clusters in understanding RV spread and in estimating the impact of interventions on RV transmission dynamics at the population level have been demonstrated. However, limitations do exist in the analysis. For instance, the deduced number (and size) of RV transmission clusters could have been underestimated because the study subjects were recruited from a single medical center. Although genetic data have been proven useful and informative in defining transmission networks, a study of such nature should be expanded to multiple recruiting locations with an extended study period, to improve the resolution and mapping of RV spatiotemporal transmission dynamics at the population level. As for the genetic distance threshold estimation, it is important to note that the current threshold was estimated based on several RV representatives with the most *VP4/VP2* sequences available for analysis. With the high genetic diversity of RV and the increasing numbers of new RV *VP4/VP2* sequences, the need to re-estimate and update the genetic distance threshold for a more reliable analysis is inevitable.

Likewise, since the protective effects of facemask, pleconaril and social distancing were previously estimated either through pseudo-steady conditions, simulations or in vitro experiments involving other viruses, the actual protective effects of these interventions on RV transmission remain elusive. However, when RV-related data are readily available, this proof-of-concept analysis may shed new insight into the effectiveness of the interventions in suppressing RV transmission at the population level.

Furthermore, current analysis focused primarily on nodes that formed transmission clusters, hence undermining the contribution of singleton in persistence and expansion of RV infection. Also, due to lack of evidence that explain the potential effects of viral factors (i.e., RV species, viral load, resistant strain), host factors (immunological response, pharmacogenetic traits) and other behavioral factors (compliance and coverage of interventions) on the RV transmission dynamics, these factors were not taken into account in our analysis. Upon availability of these information, investigations through agent-based simulation would be useful, as recently observed through 2014 Ebola outbreak [41]. In addition, future investigations should also incorporate analysis at protein level, and perhaps should garner more data from experimental work to complement findings from in silico analysis.

Although further refinement of the analysis may be necessary (when RV-related epidemiological data are readily available) to yield a real-world representation, the present study has demonstrated the resilience of genetic transmission cluster analysis in mapping transmission dynamic and estimating the potential impact of interventions on RV transmissions. Importantly, the complementary incorporation of this proof-of-concept analysis into conventional surveillance may further strengthen the epidemiological evidence, which usually limits by data incompleteness and self-reported inaccuracy.

## Conclusion

Viral genetic information is useful and robust in reconstructing the putative transmission linkages within a population with limited epidemiological data and in estimating the probable effects of interventions on viral transmission. For the first time, a network-based approach of which the degree of RV spread as well as its temporal dynamics that drive disease transmission in Kuala Lumpur, between 2012 and 2014 were mapped. A prominent clustering pattern was observed, highlighting that the RV disease burden was largely contributed by the presence of multiple sub-epidemics, driven predominantly by RV-A followed by RV-C and -B. It is important to note that no misclassifications of RV species and types were observed, highlighting the robustness of this resilient proof-of-concept analysis. In addition, the potential use of RV genetic network in assessing the impact of intervention on disease transmission was demonstrated, where social distancing is predicted to be an effective non-pharmaceutical intervention in suppressing RV transmission. The integration of such analysis into conventional epidemiological surveillance may enable the near real-time investigation of viral spread and transmission dynamics, pre-informing risk assessment for impeding disease outbreaks.

## Data Availability

Nucleotide sequences generated in the study have been deposited in GenBank under the accession numbers KY093077-KY094062. The python scripts have been made available online and accessible at https://github.com/nglj93/Transmission-Network.

## Abbreviations

COPD: Chronic obstructive pulmonary disease
COVID-19: Coronavirus disease
EP_50_: 50% Proportion of the edges
MERS-CoV: Middle East respiratory syndrome coronavirus
NP_50_: 50% Proportion of the nodes
PCR: Polymerase chain reaction
RV: Rhinovirus
RV-A: Rhinovirus A
RV-B: Rhinovirus B
RV-C: Rhinovirus C
SARS-CoV-2: Severe acute respiratory syndrome coronavirus 2
TN93: Tamura-Nei 93
